# Exploring semantic consistency in unpaired image translation to generate data for surgical applications

**DOI:** 10.1007/s11548-024-03079-1

**Published:** 2024-02-26

**Authors:** Danush Kumar Venkatesh, Dominik Rivoir, Micha Pfeiffer, Fiona Kolbinger, Marius Distler, Jürgen Weitz, Stefanie Speidel

**Affiliations:** 1 Department of Translational Surgical Oncology, National Centre for Tumor Diseases(NCT/UCC), Dresden, 01307 Germany; 2 SECAI, TU Dresden, Dresden, Germany; 3 Department of Visceral, Thoracic and Vascular Surgery, University Hospital and Faculty of Medicine, TU Dresden, 01307 Dresden, Germany; 4 The Centre for Tactile Internet(CeTI), TU Dresden, Dresden, Germany

**Keywords:** Unpaired Image translation, Semantic consistency, Laparoscopy

## Abstract

**Purpose:**

In surgical computer vision applications, data privacy and expert annotation challenges impede the acquisition of labeled training data. Unpaired image-to-image translation techniques have been explored to automatically generate annotated datasets by translating synthetic images into a realistic domain. The preservation of structure and semantic consistency, i.e., per-class distribution during translation, poses a significant challenge, particularly in cases of semantic distributional mismatch.

**Method:**

This study empirically investigates various translation methods for generating data in surgical applications, explicitly focusing on semantic consistency. Through our analysis, we introduce a novel and simple combination of effective approaches, which we call ConStructS. The defined losses within this approach operate on multiple image patches and spatial resolutions during translation.

**Results:**

Various state-of-the-art models were extensively evaluated on two challenging surgical datasets. With two different evaluation schemes, the semantic consistency and the usefulness of the translated images on downstream semantic segmentation tasks were evaluated. The results demonstrate the effectiveness of the ConStructS method in minimizing semantic distortion, with images generated by this model showing superior utility for downstream training.

**Conclusion:**

In this study, we tackle semantic inconsistency in unpaired image translation for surgical applications with minimal labeled data. The simple model (ConStructS) enhances consistency during translation and serves as a practical way of generating fully labeled and semantically consistent datasets at minimal cost. Our code is available at https://gitlab.com/nct_tso_public/constructs.

**Supplementary Information:**

The online version contains supplementary material available at 10.1007/s11548-024-03079-1.

## Introduction

The rapid advancements in deep learning (DL) techniques in the last decade has led to the growth of surgical data science [1]. However, the potential for training large and powerful models is impeded by the requirement of large annotated datasets [1, 2]. Multiple challenges contribute to this limitation, including the technical complexities in acquiring patient data directly from the operating room [3], legal regulations on data sharing, and the substantial costs involved in expert labeling, given the restricted availability of domain specialists (i.e., surgical professionals). One potential solution to overcome these challenges is adopting synthetic training data generated through computer simulations [4–6]. Synthetic data present the advantage of automatically generating substantial volumes of fully labeled data. Nonetheless, enforcing real-world characteristics in such synthetic datasets can be a significant hurdle.

**Fig. 1:**
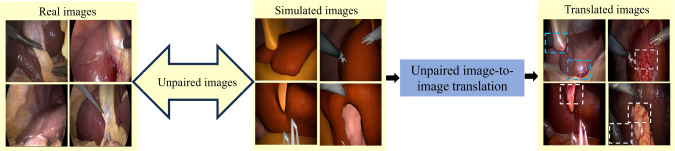
Generation of realistic data from synthetic surgical images with unpaired image translation method. The semantic mismatch between domains can lead to inconsistent translations, like blood texture (red color) getting mapped onto different structures (highlighted in white boxes). Some regions with consistent semantic translation are indicated in blue boxes

Image-to-image translation (I2I) methods are generative modeling techniques that have gained popularity for translating images between different domains. Within the field of data generation, the applicability of paired image translation methods [7] is limited. Conversely, unpaired image translation methods [8], which do not require corresponding image pairs, have emerged as promising solutions for various computer vision tasks. Overall these methods are suitable for surgical applications, but they face challenges in preserving contextual and semantic details across the domains.

In practice, translation methods aim to align the image statistics between the two domains. In addition to the difference in image distributions, semantic variations in distributions also exist, which is commonly referred to as “*unmatched semantic statistics*” [9] and poses a critical problem in preserving the semantics during translation. As displayed in Fig. 1, when faced with unmatched semantic distributions, attempting to align the distributions between translated and target images forcibly can result in spurious solutions, where semantic information is distorted [9, 10].

In real surgical scenarios, an additional challenge arises from the variations in lighting conditions, which may not be adequately reflected in existing baseline datasets [7, 11]. While synthetic images can incorporate such parameters, creating such a realistic environment takes time and effort. Also, semantic consistency can be affected when such variations exist and addressing these short comings is essential as without doing so, the generated data lacks practical utility for subsequent training of models (Section "Results").

### Our contribution

To the best of our knowledge, this study represents the first comprehensive investigation of unpaired image translation techniques to generate data in the context of surgical applications. We summarize our contribution as follows.

**Fig. 2:**
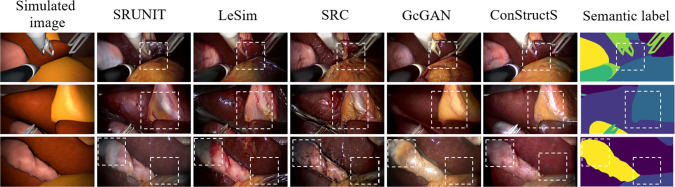
The structure and semantic characteristics of the translated images and their correspondence to the semantic labels. The ConStructS method shows consistent translation performance leading to the generation of semantically consistent dataset with labels

- We empirically analyze various methods for unpaired image translation by assessing both the semantic consistency of the translated images and their utility as training data in diverse downstream tasks.
- We tackle the underexplored problem of creating *semantic consistent datasets with annotations* (see Fig. 2). We focus on translating synthetic anatomical images into realistic surgical images on datasets from minimally-invasive surgeries, namely, cholecystectomy and gastrectomy.
- Guided by our analysis, we define a novel combination of an image quality assessment metric [12] as a loss function with the contrastive learning framework [13] as a simple yet effective modification to tackle the challenge of semantic distortion.
- We found that this simple combination to be more effective than many of the existing unpaired translation methods in maintaining semantic consistency. When the translated images from this method are mixed with the real images, we found a $22\%$ improvement in segmentation score compared to a model trained only using the real images.

## Related work

### Image-to-image translation

The objective is to generate images in a desired target domain while preserving the structure and semantics of the input. Generative adversarial networks (GANs) [14] have proven to be a powerful approach for image translation, learning the mapping between input and output images. In the case of unpaired translation, cycle consistency [8] was introduced, which seeks to learn the reverse mapping between different domains by leveraging reconstruction loss. Various approaches have been proposed to address multi-modal and domain translations, focusing on disentangling images’ content and style information in distinct spaces [4, 15–17]. In the context of the surgical application, [18] utilized cycle consistency for endoscopic image synthesis. The paired translation was adopted in [5] whereas cycle consistency with structural similarity was combined to generate laparoscopic image [4](*LapMUNIT*) and video data [6], respectively. Although these approaches effectively exploit cycle consistency, they often rely on the assumption of a bijective relationship between domains, which can be overly restrictive. Achieving perfect reconstruction becomes challenging, and they still fall behind in maintaining semantic consistency during translation.

In contrast, one-sided translation methods such as GcGAN [19], which incorporates an equivariance constraint, and DistGAN [20] enforcing consistency regularization based on distances between the images have been proposed. Efforts such as [21, 22] have been made to minimize the perceptual or content loss by utilizing a pre-trained VGG model to decrease the content disparity between the domains. However, this approach is computationally expensive and lacks adaptability to the available data. Our approach is based on a contrastive learning method proposed in CUT [13], where embeddings are learned by associating similar signals in contrast to negatives.

### Semantic robustness via losses

Despite its aim to promote content (structure) consistency, the CUT [13] method still faces challenges when the two domains have different semantic characteristics. This challenge stems from the limited ability of the contrastively learned semantics to enforce correspondence across different domains effectively. Recently, two approaches were proposed to minimize semantic distortion during translation. SRUNIT [9], based on CUT, defined a semantic robustness loss that is optimized between the input features of the domain ${\mathcal {X}}$ with the perturbated variant of the same. Similarly, a structural consistency constraint (SCC) [10] was proposed to maintain the semantics. The color randomness in the pixel values of the images before and after the translation was reduced by exploiting mutual information.

Methods like NEGCUT [23] trained a separate generator to generate negative samples dynamically, effectively bringing positive and query examples closer together, whereas F-LeSim [24] focused on preserving scene structures by minimizing losses based on spatially-correlative maps. The standalone use of any of these models fails to simultaneously reduce the domain gap and maintain semantic consistency during translation.

In this work, we devise an approach that is a novel combination of different losses, namely, the patch-based contrastive loss along with the multi-scale structural similarity [12], that regularizes the model on various image resolutions, thereby maintaining consistent translations between the simulated and realistic domains. This approach relies neither on cycle consistency nor other additional networks during translation, thereby paving the way for one-sided, unpaired image translation. Many of the stated approaches have focused primarily on just realism as the central concept during translation. However, for surgical application in hand, it is equally important to access both the semantic consistency and the usefulness of such translated images in downstream applications.

## Model setup

In this section, we provide an overview of the essential components for the formulation of the approach that preserves both the content and semantic correlation between the anatomical structures during translation.

### Adversarial learning

GANs [14] have been promising candidates for image translation tasks. The main goal of such an image translation technique is to acquire the ability to map between two domains, ${\mathcal {X}}$ and ${\mathcal {Y}}$, based on training samples ${x_i}$ and ${y_j}$ drawn from the distributions *p*(*X*) and *p*(*Y*), respectively. The generator $G_{\mathcal{X}\mathcal{Y}}$ learns the mapping between domains and generates the translated image ${\mathcal {T}}(y)$ and the discriminator $D_{{\mathcal {Y}}}$ is trained to distinguish between original images *x* and translated images. The adversarial loss is defined as,

1 $$\begin{aligned}&{\mathcal {L}}_{G A N}\left( G_{\mathcal{X}\mathcal{Y}}, D_{{\mathcal {Y}}}\right) ={\mathbb {E}}_{y \sim p(Y)}\left[ \log D_{{\mathcal {Y}}}(y)\right] \nonumber \\&\quad +{\mathbb {E}}_{x \sim p(X)}\left[ \log \left( 1-D_{{\mathcal {Y}}}\left( G_{\mathcal{X}\mathcal{Y}}(x)\right) \right) .\right. \end{aligned}$$

Typically, the loss is used to encourage the distributional match between the translated images and images from domain ${\mathcal {Y}}$.

### Patch constrastive learning

This framework was formulated on noise contrastive estimation (NCE), aiming to maximize the mutual information between the domains. The InfoNCE loss [25] was used to learn embeddings between the domains and establish associations between corresponding patches of input and output images while disassociating them if unrelated. The central idea lies in associating a “query” point with the “positive” points while contrasting away from other “negative” points in the dataset. Let *s* be the query vector and $s^{+}$ and $s^{-}$ be the positive and negative vectors from the images, respectively. The $s^{-}$ vectors are sampled at *N* different locations in the input. Finally, the loss is formulated as an (N+1)- way classification and defined as

2 $$\begin{aligned} {\mathcal {L}}_{NCE} = -\log \left[ \frac{\exp \left( {\varvec{s}} \cdot {\varvec{s}}^{+} / \tau \right) }{\exp \left( {\varvec{s}} \cdot {\varvec{s}}^{+} / \tau \right) +\sum _{n=1}^N \exp \left( {\varvec{s}} \cdot {\varvec{s}}_n^{-} / \tau \right) }\right] \nonumber \\ \end{aligned}$$

where $\tau$ is a scaling parameter to factor the distances between the vectors. The query vector is drawn from the translated images, while $s^{+}$ and $s^{-}$ are the corresponding and non-corresponding image (feature) vectors from the input images. We refer to the suppl. material for the computation procedure of these vectors.

A multilayer patch-based contrastive loss was further employed within the CUT framework, formally defined as *PatchNCE*. It leverages the ready availability of the generator $G_{\mathcal{X}\mathcal{Y}}$ to extract features from *L* layers at *S* spatial locations. The *PatchNCE* loss is defined as,

3 $$\begin{aligned} {\mathcal {L}}_{\textrm{Patch}}(X)={\mathbb {E}}_{{\varvec{x}} \sim X} \sum _{l=1}^L \sum _{s=1}^{S} {\mathcal {L}}_{NCE} \end{aligned}$$

### Semantic consistency

Next, we define the *multi-scale structural similarity* (MS-SSim) [12] metric. This measure was proposed as a metric for image quality assessment. The extracted structure information from the images is compared on varying image resolutions with a weighting factor for each. Initially, given two images, ${\textbf{x}}$ and ${\textbf{y}}$, let $v_1 = 2 \sigma _{xy} + C_2$ and $v_2 = \sigma _{x}^{2} + \sigma _{y}^{2} + C_2$. Then contrast sensitivity($\textbf{cs}$) and structure map ($\textbf{ss}$) are defined as,

4 $$\begin{aligned} {\text {cs}}({\textbf{x}},{\textbf{y}}) = \frac{v_1}{v_2}, \quad {\text {ss}}({\textbf{x}},{\textbf{y}}) = \frac{(2 \mu _{x} \mu _{y} + C_1) v_1}{(\mu _{x}^{2} + \mu _{y}^{2} + C_1) v_2} \end{aligned}$$

where $\mu _{(\cdot )}$ and $\sigma _{(\cdot )}$ are the mean and variance of the image(pixels) and $\sigma _{x,y}$ is the covariance between ${\textbf{x}}$ and ${\textbf{y}}$. $C_1$ and $C_2$ are stability constants computed as $(K*L)^2$ and $K \ll 1$, *L* depending on the dynamic range of pixels ($0-255$). The MS-SSim metric is defined as,

5 $$\begin{aligned} {\text {MS-SSim}}({\textbf{x}}, {\textbf{y}})=\left[ W_i\right] \cdot \prod _{i=1}^K\textbf{cs}_i \cdot \textbf{ss}_i \end{aligned}$$

where $i=1\cdots K$ denotes the number of different image scales and $W_i$ the weight for the *i*th scale. Hereafter, we mention the loss as *semantic loss*. It is defined as,

6 $$\begin{aligned} {\mathcal {L}}_{\textrm{semantic}} = 1 - {\text {MS-SSim}}(x,y) \end{aligned}$$

#### Constrastive learning coupled with MS-SSim

We couple the strengths of both Contrastive Learning with Structural Similarity (ConStructS) as a model to tackle semantic distortion. To the best of our knowledge, this combination has not been proposed yet. As a combined loss the image features at patch level are learned to enforce correspondences during translation. The final objective is defined as,

7 $$\begin{aligned} {\mathcal {L}}_{\textrm{total}} = {\mathcal {L}}_{GAN} {+} \lambda _{x} {\mathcal {L}}_{\textrm{Patch}}(X) {+} \lambda _{y} {\mathcal {L}}_{\textrm{Patch}}(Y) {+} \lambda _{ss} {\mathcal {L}}_{\textrm{semantic}}\nonumber \\ \end{aligned}$$

where $\lambda _x$, $\lambda _y$, and $\lambda _{ss}$ are weighting parameters for the PatchNCE and semantic losses, respectively. The ${\mathcal {L}}_{Patch}(Y)$ resembles the identity loss [8] and is applied between the images $y \in {\mathcal {Y}}$ and translated images. The ConStructS approach is a one-sided unpaired translation method that relies on no additional generators or discriminators and imposing the ${\mathcal {L}}_{\textrm{Patch}}(Y)$ component is necessary to prevent degenerate cases from the generator.

## Experiments

In this section, we outline our experiments where the performance of several popular unpaired image translation models are compared. The models include CycleGAN [8], the VGG-based perpetual loss [22], DRIT${++}$ [26], LapMUNIT [4], UGAT-IT [27] using cycle consistency, one-sided approach such as GcGAN [19] and DistGAN [20] Also, various configurations of contrastive-based models were investigated. The CUT model was trained with the SCC loss [10] and SRC [28]. F/LeSim [24], SRUNIT [9] and NEGCUT [23] were trained with the CUT as the backbone. We demonstrate the effectiveness of ConStructS in translating synthetic data to the realistic domain with minimal semantic distortion. In particular, the existing baselines exhibit distinct strengths and weaknesses. While certain baselines excel in specific tasks, they may falter in others. Except for LapMUNIT [4] and CycleGAN [8], no tailored approach exists for surgical scenarios. Consequently, we evaluate ConStructS against several other methods to align with the prevailing research.

Finally, we provide a rationale for the design choices made in the ConStructS model to ensure semantic consistency with an ablation study. We train the model without the semantic loss, which reverts to the basic CUT model [13] and without the *PatchNCE* loss. Similarly, we combined the *semantic* loss with cycle consistency into the CycleGAN model for a different combination. For the details on implementation the readers can be refer to the suppl. material.

### Data

We evaluated the methods mentioned above on two different surgical datasets consisting of anatomical organs such as the liver, liver ligament, gallbladder, abdominal wall, pancreas as well as surgical tools.

#### Cholecystectomy dataset

This surgery serves to remove the gallbladder. For the simulated domain ${\mathcal {X}}$, we utilized the publicly available synthetic dataset resembling laparoscopic scenes [4]. A total of 20, 000 rendered images forms the synthetic dataset. The real images for the domain ${\mathcal {Y}}$ are taken from the Cholec80 data set [29]. We finally created a training dataset of approximately 26, 000 images from 75 patients. A separate segmentation dataset of 5 patients was chosen. The liver was manually segmented in 196 images for the downstream evaluation (Sect. 4.2.2). The images were cropped to 256 x 512 pixels, and the training set consists of 17, 500 images, with the remaining 2500 serving as the test set.

#### Gastrectomy dataset

For this case, we utilized the real and synthetic dataset from [5], based on 40 real surgical videos of distal gastrectomy. The dataset consists of 3400 synthetic and 4500 real images with corresponding segmentation masks. 2400 images constituted the training set, with 1000 images as the test set. The images were resized and cropped to 512 x 512 pixels.

### Evaluation

We adopted two different schemes to assess both the semantic consistency and the usefulness of generating such data.

#### Train:Real $\to$Eval:Synthetic

Firstly, we adopted the practice of computing metrics based on an *off-the-shelf* segmentation model following [5, 11, 13, 19]. We train a segmentation model on the real images of the specific dataset. Then the translated synthetic images are tested using this pre-trained model i.e, the metrics are computed against ground truth labels of the synthetic images. The underlying intuition is that, if the translation model is able to reduce the domain gap, then the segmentation accuracy from this pre-trained model on the translated synthetic images would be higher [7]. This method assesses both the quality, as well as semantic consistency of the translated images. We refer to this method as *consistency* evaluation.

#### Translated images as training data

Furthermore, we assess the practical utility of the translated images in a downstream task in two different methods. Firstly, we train a segmentation model using only the translated images and evaluate the performance of this model on segmenting the organ liver on real images. Secondly, we fine-tune this model on the real data and evaluate them on the same test set of real images. The performance is also compared to a baseline model trained only on real images. This approach aligns with the intuition mentioned above and provides insights into the realism of the translated images. We report the mean dice scores for this method. Hereafter, we refer to this method as *downstream* evaluation.

**Table 1:** *Consistency* evaluation results of various translation models on the cholecystectomy dataset. pxAcc and clsAcc denotes the pixel and mean class accuracy, respectively. mIOU is the mean intersection over union scores

Approach	Method	pxAcc	clsAcc	mIOU
Cycle consistency	CycleGAN [8]	$0.49\pm 0.08$	$0.41\pm 0.14$	$0.23\pm 0.09$
	CycleGAN+VGG [22]	$0.52\pm 0.09$	$0.43\pm 0.11$	$0.25\pm 0.10$
	DRITT$++$ [26]	$0.42\pm 0.03$	$0.28\pm 0.05$	$0.17\pm 0.04$
	LapMUNIT [4]	${\underline{0.53\pm 0.06}}$	$0.38\pm 0.08$	$0.25\pm 0.06$
	UGAT-IT [27]	$0.40\pm 0.03$	$0.28\pm 0.05$	$0.16\pm 0.04$
One-sided translation	GcGAN [19]	$0.51\pm 0.08$	${\textbf{0}}.{\textbf{44}}\pm {\textbf{0}}.{\textbf{10}}$	$0.26\pm 0.08$
	DistGAN [20]	$0.40\pm 0.03$	$0.28\pm 0.50$	$0.16\pm 0.04$
Contrastive learning	SRC [28]	$0.51\pm 0.07$	${\underline{0.43\pm 0.16}}$	$0.25\pm 0.09$
	NEGCUT [23]	$0.49\pm 0.08$	$0.41\pm 0.15$	$0.23\pm 0.09$
	FeSim [24]	$0.41\pm 0.10$	$0.37\pm 0.16$	$0.20\pm 0.09$
	LeSim [24]	$0.47\pm 0.09$	$0.43\pm 0.13$	$0.24\pm 0.09$
Semantic consistency	CycleGAN$+$SCC [10]	$0.50\pm 0.10$	$0.43\pm 0.15$	$0.25\pm 0.10$
	CUT$+$SCC [10]	$0.42\pm 0.06$	$0.35\pm 0.12$	$0.18\pm 0.07$
	SRUNIT [9]	$0.50\pm 0.08$	$0.40\pm 0.13$	$0.23\pm 0.08$
Ablation study	ConStructS w/o ${\mathcal {L}}_{\textrm{semantic}}$ [13]	$0.50\pm 0.07$	$0.40\pm 0.14$	${\underline{0.26\pm 0.09}}$
	ConStructS w/o PatchNCE	$0.50\pm 0.10$	$0.40\pm 0.14$	$0.25\pm 0.10$
	CycleGAN$+$ ${\mathcal {L}}_{\textrm{semantic}}$	$0.49\pm 0.10$	$0.43\pm 0.15$	$0.24\pm 0.09$
	ConStructS	${\textbf{0}}.{\textbf{59}}\pm {\textbf{0}}.{\textbf{07}}$	${\textbf{0}}.{\textbf{44}}\pm {\textbf{0}}.{\textbf{12}}$	${\textbf{0}}.{\textbf{29}}\pm {\textbf{0}}.{\textbf{09}}$

The best result is indicated in **bold**, and the second best is underlined

### Results

#### Cholecystectomy dataset

The quantitative results are presented in Table 1, highlighting the performance of different models. Comparatively, the CycleGAN model with the VGG loss demonstrates better performance than SCC variant. The geometric consistency in GcGAN [19] leads to a comparable class-accuracy value with ConStructS while outperforming DistGAN [20] and DRIT++ [26]. The LapMUNIT [4] model achieves better scores than the attention-based models. As for the variants of CUT, the addition of SCC loss did not improve its performance further. Overall, as evidenced by the results, the ConStructS model minimizes semantic distortion to a greater extent and outperforms the recent methods.

**Table 2:** The quantitative results (mean dice scores) for *downstream* eval. Pretraining followed by fine-tuning on real images leads to considerable performance gain using images from the ConStructS method

Data generation method	Syn	Syn+Real
CycleGAN [8]	$0.62\pm 0.14$	$0.74\pm 0.10$
GcGAN [19]	$0.63\pm 0.14$	$0.78\pm 0.05$
LapMUNIT [4]	$0.56\pm 0.19$	$0.68\pm 0.16$
SRUNIT [9]	$0.61\pm 0.13$	$0.75\pm 0.07$
ConStructS (ours)	${\textbf{0}}.{\textbf{65}}\pm {\textbf{0}}.{\textbf{21}}$	${\textbf{0}}.{\textbf{84}}\pm {\textbf{0}}.{\textbf{05}}$
Baseline (real data only)	$0.62\pm 0.11$	

Bold values indicate the best results

**Fig. 3:**
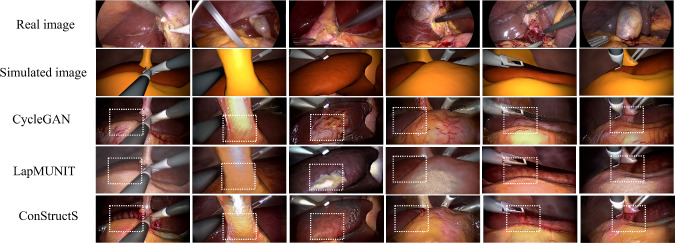
Qualitative results of various translation methods on the cholecystectomy dataset. At the junction of two structures, the textures were interchanged in most of the models. Although not solved completely, the ConStructS model reduces semantic inconsistency. Some regions are highlighted in white boxes

Table 2 indicates the results of the *downstream* evaluation. When only the translated synthetic images are used as training data, the ConStructS model yields comparable results on segmenting the liver to GcGAN [19]. A gain of $3\%$ in dice score is obtained compared to the baseline model. Fine-tuning the same model on real data shows that the ConStructS method outperforms all the models, showing an improvement of $22\%$ compared to the baseline. The qualitative results in Fig. 3 indicate that the ConStructS model reduces the semantic distortion, although not completely, but better than most other translation methods.

#### Gastrectomy dataset

As presented in Table 3, quantitative analysis reveals that LapMUNIT [4] outperforms both GcGAN [19] and CycleGAN [8] models. Conversely, the ConStructS model significantly mitigates semantic mismatches and exhibits a moderate improvement in performance compared to all the other models. Readers can refer to suppl. material for additional results.

#### Ablation study

**Fig. 4:**
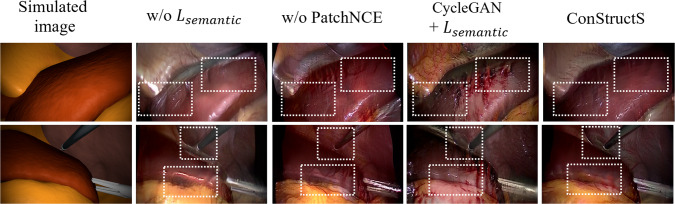
Qualitative results of the ablation study on the cholecystectomy dataset. Texture mismatch occurs in low-lighting regions without the semantic loss. As seen from the 2nd row without the PatchNCE loss, no explicit boundary exists between the liver and abdominal wall leading to both regions having the same semantic textures

The qualitative results of the ablation study are presented in Fig. 4. When examining the CUT model, specifically ConStructS, without semantic loss, we observe that the structure is well preserved during translation. However, there is a noticeable mismatch in texture in regions with reduced brightness. In the absence of the PatchNCE loss, as there is no explicit control over image patches, structure information is mixed, resulting in the different style mapping (e.g., fat or blood) to unlikely structures. Lastly, the combination of the semantic loss with the CycleGAN model yields an improvement compared to the basic CycleGAN model. Regardless, as seen from Table 1, this combination still lacks performance.

**Table 3:** The quantitative results of the *consistency* eval. on the gastrectomy dataset

Method	pxAcc	clsAcc	mIOU
CycleGAN [8]	$0.39\pm 0.12$	$0.17\pm 0.14$	$0.09\pm 0.10$
GcGAN [19]	$0.40\pm 0.13$	$0.18\pm 0.01$	$0.10\pm 0.01$
LapMUNIT [4]	$0.43\pm 0.01$	$0.21\pm 0.10$	$0.11\pm 0.09$
CUT [13]	$0.42\pm 0.01$	${\underline{0.22\pm 0.02}}$	${\textbf{0}}.{\textbf{11}}\pm {\textbf{0}}.{\textbf{05}}$
SRUNIT [9]	${\underline{0.44\pm 0.01}}$	$0.20\pm 0.01$	$0.10\pm 0.05$
ConStructS	${\textbf{0}}.{\textbf{46}}\pm {\textbf{0}}.{\textbf{08}}$	${\textbf{0}}.{\textbf{24}}\pm {\textbf{0}}.{\textbf{13}}$	${\underline{0.10\pm 0.09}}$

The best result is indicated in **bold**, and the second best is underlined

### Discussion

Traditional approaches, such as DistGAN [20] or the $L_1$ reconstruction loss in CycleGAN [8], typically do not effectively enhance semantic consistency. They are susceptible to structural transformations and variations in lighting conditions, which can introduce artifacts during translation (Fig. 3). While SRUNIT [9] and CUT [13] show promise in reducing semantic distortion, they alone are insufficient for the surgical application. On the contrary, the NEGCUT [23] model aims to preserve the overall structure during translation but needs to be more accurate in mapping textures between these structures. The same limitation has been observed in the LeSim [24] model. Although LapMUNIT [4] utilizes the semantic loss with cycle consistency, semantic inconsistency still prevails and is reflected in the results (Fig. 3). Enforcing the perceptual loss [22] with additional networks did not improve performance.

The results of our ablation study demonstrate the crucial role of combining PatchNCE with semantic loss in mitigating semantic distortion. We posit that leveraging the contrastive learning approach makes learning higher-level attributes, such as organ or tool structures, possible. However, relying solely on this aspect for matching semantic information has limitations [9]. To address this, we introduced the semantic loss as a regularizer that operates on the multiple scales of the images (i.e., different resolutions). This loss additionally checks the images’ perceptual quality, factoring the challenging lighting conditions (Eq. 4). This combination of losses proves effective in preserving the semantic characteristics throughout the translation process.

#### Limitations

The ConStructS model holds promise for mitigating semantic inconsistencies; however, it is essential to acknowledge its limitations. Notably, this method overlooks the synthesis of multi-modal data. By incorporating multi-model outcomes with additional apriori information (such as segmentation mask), this model emerges as a promising candidate for generating structure-specific and diverse surgical images. Additionally, adding per-frame consistency leads to generating temporally consistent surgical video datasets. As a future line of work, we believe ConStructS to be a valuable model to address the challenges in developing annotated video datasets.

## Conclusion

In conclusion, we conducted an empirical investigation on the issue of semantic inconsistency in unpaired image translation, focusing on its relevance to surgical applications where labeled data are minimal. We extensively evaluate several state-of-the-art unpaired translation methods, explicitly targeting the translation of images from a simulated domain to a realistic environment. Addressing the problem of semantic distortion, we found a novel combination of a structure similarity metric with contrastive learning as the most effective. Surprisingly, this simple model reduces semantic distortion while preserving the realism of the translated images and shows the highest utility as training data for downstream tasks.

## Supplementary Information

Below is the link to the electronic supplementary material.

Supplementary file 1 (pdf 20644 KB)

## References

[1] Maier-Hein L, Eisenmann M, Sarikaya D, März K, Collins T, Malpani A, Fallert J, Feussner H, Giannarou S, Mascagni P, Nakawala H, Park A, Pugh C, Stoyanov D, Vedula SS, Cleary K, Fichtinger G, Forestier G, Gibaud B, Grantcharov T, Hashizume M, Heckmann-Nötzel D, Kenngott HG, Kikinis R, Mündermann L, Navab N, Onogur S, Roß T, Sznitman R, Taylor RH, Tizabi MD, Wagner M, Hager GD, Neumuth T, Padoy N, Collins J, Gockel I, Goedeke J, Hashimoto DA, Joyeux L, Lam K, Leff DR, Madani A, Marcus HJ, Meireles O, Seitel A, Teber D, Ückert F, Müller-Stich BP, Jannin P, Speidel S (2022) Surgical data science-from concepts toward clinical translation. Med Image Anal 76:102306 34879287 10.1016/j.media.2021.102306 PMC9135051

[2] Maier-Hein L, Vedula SS, Speidel S, Navab N, Kikinis R, Park A, Eisenmann M, Feussner H, Forestier G, Giannarou S, Hashizume M, Katic D, Kenngott H, Kranzfelder M, Malpani A, März K, Neumuth T, Padoy N, Pugh C, Schoch N, Stoyanov D, Taylor R, Wagner M, Hager GD, Jannin P (2017) Surgical data science for next-generation interventions. Nat Biomed Eng 1(9):691–696 31015666 10.1038/s41551-017-0132-7

[3] Hager GD, Maier-Hein L, Vedula SS (2020) Chapter 38 - surgical data science. In: Zhou SK, Rueckert D, Fichtinger G (eds) Handbook of medical image computing and computer assisted intervention. The Elsevier and MICCAI society book series. Academic Press, pp 931–952

[4] Pfeiffer M, Funke I, Robu MR, Bodenstedt S, Strenger L, Engelhardt S, Roß T, Clarkson MJ, Gurusamy K, Davidson BR, Maier-Hein L, Riediger C, Welsch T, Weitz J, Speidel S (2019) Generating large labeled data sets for laparoscopic image processing tasks using unpaired image-to-image translation. In: Medical image computing and computer assisted intervention–MICCAI 2019: 22nd international conference, Shenzhen, China, October 13–17, 2019, Proceedings, Part V 22, pp. 119–127. Springer

[5] Yoon J, Hong S, Hong S, Lee J, Shin S, Park B, Sung N, Yu H, Kim S, Park S, Hyung WJ, Choi M-K (2022) Surgical scene segmentation using semantic image synthesis with a virtual surgery environment. In: Medical image computing and computer assisted intervention–MICCAI 2022: 25th international conference, Singapore, September 18–22, 2022, Proceedings, Part VII, pp. 551–561. Springer

[6] Rivoir D, Pfeiffer M, Docea R, Kolbinger F, Riediger C, Weitz J, Speidel S (2021) Long-term temporally consistent unpaired video translation from simulated surgical 3d data. In: Proceedings of the IEEE/CVF international conference on computer vision, pp. 3343–3353

[7] Isola P, Zhu J-Y, Zhou T, Efros AA (2017) Image-to-image translation with conditional adversarial networks. In: Proceedings of the IEEE conference on computer vision and pattern recognition, pp. 1125–1134

[8] Zhu J-Y, Park T, Isola P, Efros AA (2017) Unpaired image-to-image translation using cycle-consistent adversarial networks. In: Proceedings of the IEEE international conference on computer vision, pp. 2223–2232

[9] Jia Z, Yuan B, Wang K, Wu H, Clifford D, Yuan Z, Su H (2021) Semantically robust unpaired image translation for data with unmatched semantics statistics. In: Proceedings of the IEEE/CVF international conference on computer vision, pp. 14273–14283

[10] Guo J, Li J, Fu H, Gong M, Zhang K, Tao D (2022) Alleviating semantics distortion in unsupervised low-level image-to-image translation via structure consistency constraint. In: Proceedings of the IEEE/CVF conference on computer vision and pattern recognition, pp. 18249–18259

[11] Chu C, Zhmoginov A, Sandler M (2017) Cyclegan, a master of steganography. arXiv preprint arXiv:1712.02950

[12] Wang Z, Simoncelli EP, Bovik AC (2003) Multiscale structural similarity for image quality assessment. In: The thrity-seventh asilomar conference on signals, systems & computers, 2003, vol. 2, pp. 1398–1402. IEEE

[13] Park T, Efros AA, Zhang R, Zhu J-Y (2020) Contrastive learning for unpaired image-to-image translation. In: Computer vision–ECCV 2020: 16th European conference, Glasgow, UK, August 23–28, 2020, proceedings, Part IX 16, pp. 319–345. Springer

[14] Goodfellow I, Pouget-Abadie J, Mirza M, Xu B, Warde-Farley D, Ozair S, Courville A, Bengio Y (2020) Generative adversarial networks. Commun ACM 63(11):139–144

[15] Liu M-Y, Breuel T, Kautz J (2017) Unsupervised image-to-image translation networks. Adv Neural Inform Process Syst **30**

[16] Huang X, Liu M-Y, Belongie S, Kautz J (2018) Multimodal unsupervised image-to-image translation. In: Proceedings of the European conference on computer vision (ECCV), pp. 172–189

[17] Zhu J-Y, Zhang R, Pathak D, Darrell T, Efros AA, Wang O, Shechtman E (2017) Toward multimodal image-to-image translation. Adv Neural Inform Process Syst **30**

[18] Sharan L, Romano G, Koehler S, Kelm H, Karck M, De Simone R, Engelhardt S (2021) Mutually improved endoscopic image synthesis and landmark detection in unpaired image-to-image translation. IEEE J Biomed Health Inform 26(1):127-138 10.1109/JBHI.2021.3099858 34310335

[19] Fu H, Gong M, Wang C, Batmanghelich K, Zhang K, Tao D (2019) Geometry-consistent generative adversarial networks for one-sided unsupervised domain mapping. In: Proceedings of the IEEE/CVF conference on computer vision and pattern recognition, pp. 2427–2436 10.1109/cvpr.2019.00253 PMC7030214 32076365

[20] Tran N-T, Bui T-A, Cheung N-M (2018) Dist-gan: an improved gan using distance constraints. In: Proceedings of the European conference on computer vision (ECCV), pp. 370–385

[21] Dosovitskiy A, Brox T (2016) Generating images with perceptual similarity metrics based on deep networks. Adv Neural Inform Process Syst **29**

[22] Johnson J, Alahi A, Fei-Fei L (2016) Perceptual losses for real-time style transfer and super-resolution. In: Computer vision–ECCV 2016: 14th European conference, Amsterdam, The Netherlands, October 11-14, 2016, Proceedings, Part II 14, pp. 694–711. Springer

[23] Wang W, Zhou W, Bao J, Chen D, Li H (2021) Instance-wise hard negative example generation for contrastive learning in unpaired image-to-image translation. In: Proceedings of the IEEE/CVF international conference on computer vision, pp. 14020–14029

[24] Zheng C, Cham T-J, Cai J (2021) The spatially-correlative loss for various image translation tasks. In: Proceedings of the IEEE/CVF conference on computer vision and pattern recognition, pp. 16407–16417

[25] Oord Avd, Li Y, Vinyals O (2018) Representation learning with contrastive predictive coding. arXiv preprint arXiv:1807.03748

[26] Lee H-Y, Tseng H-Y, Huang J-B, Singh M, Yang M-H (2018) Diverse image-to-image translation via disentangled representations. In: Proceedings of the European conference on computer vision (ECCV), pp. 35–51

[27] Kim J, Kim M, Kang H, Lee K (2019) U-gat-it: unsupervised generative attentional networks with adaptive layer-instance normalization for image-to-image translation. arXiv preprint arXiv:1907.10830 10.3390/s23156858 PMC10422294 37571641

[28] Jung C, Kwon G, Ye JC (2022) Exploring patch-wise semantic relation for contrastive learning in image-to-image translation tasks. In: Proceedings of the IEEE/CVF conference on computer vision and pattern recognition, pp. 18260–18269

[29] Twinanda AP, Shehata S, Mutter D, Marescaux J, De Mathelin M, Padoy N (2016) Endonet: a deep architecture for recognition tasks on laparoscopic videos. IEEE Transact Med Imaging 36(1):86–97 10.1109/TMI.2016.2593957 27455522

