# Supplementary material for: Exploring semantic consistency in unpaired image translation to generate data for surgical applications
Source: Int J Comput Assist Radiol Surg. 2024 Feb 26;19(6):985–93. doi: 10.1007/s11548-024-03079-1 (PMC11599420; doi:10.1007/s11548-024-03079-1)
Supplement: Supplementary file 1 — (pdf 20644 KB) [file 11548_2024_3079_MOESM1_ESM.pdf]

Supplementary Material: Exploring Semantic Consistency in Unpaired Image Translation to Generate Data for Surgical Applications

Danush Kumar Venkatesh[1,2,3*], Dominik Rivoir[1,4], Micha Pfeiffer[1], Fiona Kolbinger[1,3], Marius Distler[3], Jürgen Weitz[3,4], Stefanie Speidel[1,2,3,4]

[1*]Department of Translational Surgical Oncology, National Centre for Tumor Diseases, Dresden, 01307, Germany.
[2]SECAI, TU Dresden, Germany.
[3]Department of Visceral, Thoracic & Vascular Surgery, University Hospital & Faculty of medicine, TU Dresden, 01307, Germany.
[4]The Centre for Tactile Internet(CeTI), TU Dresden, Germany.

*Corresponding author(s). E-mail(s): danushkumar.venkatesh@nct-dresden.de;

1 Dataset

For the cholecystectomy dataset, the liver meshes were taken from a public dataset (3D-IRCADb 01 data set, IRCAD, France), while all other structures were designed manually. The camera was moved around along with the light source of the laparoscope, and the synthetic images were rendered. For the real dataset, the images were extracted at a frame rate of five frames per second. A total of 75 videos were chosen and the images were then curated to remove scenes with only anatomical structures or tools, and finally, a dataset of $26,000$ images was composed. The remaining 5 videos were chosen fro the *downstream* evaluation. The synthetic dataset has been downloaded from http://opencas.dkfz.de/image2image/. Similarly, for the gastrectomy dataset, the entire dataset along with labels has been downloaded from https://www.kaggle.com/datasets/yjh4374/sisvse-dataset. Figure 1 shows some examples of the dataset.

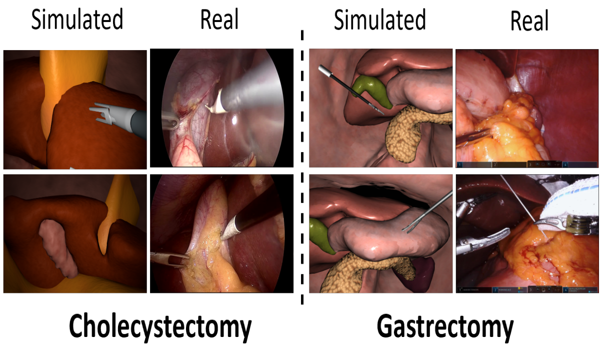

Fig. 1 Examples from both the surgical datasets.

2 Training details

The architectures and hyperparameters were intentionally matched to have a reasonable performance comparison to the CUT [1] and CycleGAN [2] models and their variants. Following [3], the semantic loss was operated on the images' brightness (average over the channels) as this retains the brightness variations while avoiding the penalization of style-related changes in hue.

For the ConStructS model, we used a discriminator similar to CUT [1] but replaced the normalization layers with the spectral norm [4] for stabilized training. The Adam optimizer [5] was utilized with a learning rate of $2e{-}4$ with a linear decay in learning rate. The generator also serves the purpose of the feature encoder to compute the contrastive loss. Correspondingly, the features were encoded from the $1, 4, 8, 12$ and 16th layer of the generator. This was constantly maintained for both datasets. The encoded features are passed through a two-layer MLP with 256 neurons each to extract the feature vectors and are normalized with the $L2$ norm. These feature vectors were utilized for computing the *PatchNCE* loss at 256 different locations. A batch size of 1 was employed throughout. For the cholecystectomy dataset, the model was trained for approximately 600K iterations, whereas for the gastrectomy dataset, the training was carried out until 500K iterations. The models were trained on single NVIDIA RTX A5000 GPUs with 24GB memory.

All the baseline models were trained based on author's code as mentioned below,

- CycleGAN [2]: https://github.com/junyanz/pytorch-CycleGAN-and-pix2pix
- GcGAN [6]: https://github.com/hufu6371/GcGAN/tree/master
- DistanceGAN [7]: https://github.com/sagiebenaim/DistanceGAN/tree/master
- DRIT++ [8]: https://github.com/HsinYingLee/DRIT/
- LapMUNIT [3]: https://gitlab.com/nct_tso_public/laparoscopic-image-2-image-translation
- UGAT-IT [9]: https://github.com/znxlwm/UGATIT-pytorch
- NEGCUT [10]: https://github.com/WeilunWang/NEGCUT
- F/LeSim [11]: https://github.com/lyndonzheng/F-LSeSim
- SCC [12]: https://github.com/CR-Gjx/SCC

- SRC [13]: https://github.com/jcy132/Hneg_SRC
- SRUNIT [14]: https://github.com/SeanJia/SRUNIT
- Perceptual loss [15]: https://github.com/dxyang/StyleTransfer
- CUT [1]: https://github.com/taesungp/contrastive-unpaired-translation

The same image size was maintained throughout for each dataset during training of all the baseline models.

3 Evaluation details

For the cholecystectomy dataset, the CholecSeg8K dataset [16], which is an annotated subset of real images from the Cholec 80 dataset, was used for training. The training dataset consists of 6000 images with a test set of 2020 images. The DeepLabV3+ [17] model was chosen as the segmentation network. We curated the dataset and defined six classes, namely, the liver, abdominal wall, fat, ligament, gallbladder, surgical tools, and background. The evaluation was posed as a multiclass segmentation problem. The different partitions of the tools were fused together into a single tool class. Similarly, for the gastrectomy dataset, six classes were defined: surgical tools, liver, stomach, spleen, pancreas, and gallbladder. Following [18], the models were trained on three different folds of train and test datasets.

For the *downstream* eval. method, five separate videos were chosen from the Cholec80 dataset. These videos were chosen such that they were not present in the CholecSeg8K dataset. The translation models are not exposed these images during training. Since annotating all the tissues and tools would require the guidance of a medical professional and, to simplify the process, only the liver tissue was annotated. The labelme [19] package was used to manually annotate the liver tissue in 196 images from five different patients. The regions of the liver with minimal lighting were under-segmented in case of doubt to ease the annotation process. A similar DeepLabV3+ model was employed to classify the liver organ. The models were trained and evaluated in leave-one-patient-out method i.e., the model is trained on four patients and evaluated on one patient and this procedure is followed five times. Finally, the mean dice scores is reported. The Adam optimizer was used along with a learning rate of $1e{-}5$. The Onecycle [20] scheduler was used to modulate the learning rate during training.

4 Sensitivity analysis.

In this section, we study the sensitivity of the parameter λ_{SS} and the direct influence of the *semantic* loss. The parameter λ_{SS} is varied between the values $1, 2, 3, 5$ and 10. As the λ_{SS} value is increased, there is a performance improvement up to a certain threshold. Figure 2 and Table 1 indicate that setting the appropriate λ_{SS} (here, 5) effectively controls the irregular texture between the gallbladder and the tool. However, it is necessary to note that large values limit the benefits of the semantic loss, as the model primarily focuses on reducing structure distortion and disregards the style information. A binary search could identify the largest λ_{SS} value that maintains the semantic character. Some additional results are shown in Figure 3.

λ_{SS}	pxAcc	clsAcc	mIOU
1	0.50 ± 0.06	0.42 ± 0.14	0.24 ± 0.08
2	0.52 ± 0.07	0.43 ± 0.14	0.25 ± 0.09
3	0.54 ± 0.06	0.43 ± 0.13	0.25 ± 0.08
5	0.59 ± 0.07	0.44 ± 0.12	0.29 ± 0.09
10	0.56 ± 0.08	0.42 ± 0.13	0.27 ± 0.09

Table 1 The *consistency* eval. results of the sensitivity analysis on λ_{SS} for the cholecystectomy dataset.

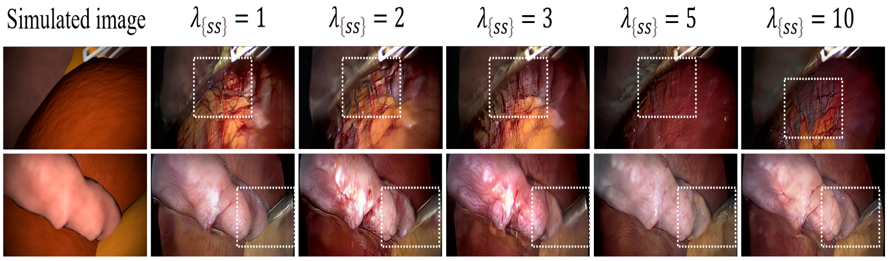

Fig. 2 Sensitivity analysis examples on the cholecystectomy dataset. For the λ_{SS} value being 5, liver texture is maintained (1st row) and the tool texture (grey lining) is avoided between the junction of the structures (2nd row).

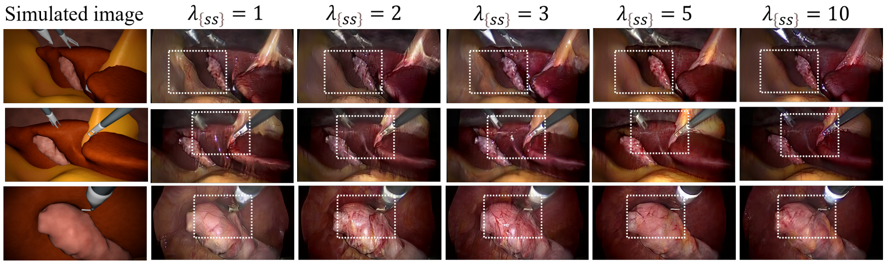

Fig. 3 Additional results from the sensitivity analysis on the cholecystectomy dataset. The structure of the surgicall tool is the best maintained with $\lambda_{SS} = 5$. Similarly, for the same value we find that blood texture is not mixed with either the liver organ or the junction between the ligament and liver.

5 Additional results

5.1 Cholecystectomy

We provide additional qualitative results for both datasets. The Figure 4 further indicates that the ConStructS model is able to maintain both structure and semantic consistency when compared to many of the other models. Furthermore, the additional results of the ablation study from Figure 5 show the importance of combining the *PatchNCE* loss with the *semantic* loss to reduce semantic distortion. Table 2 indicates the results for an ablation study where the ConStructS method is trained with

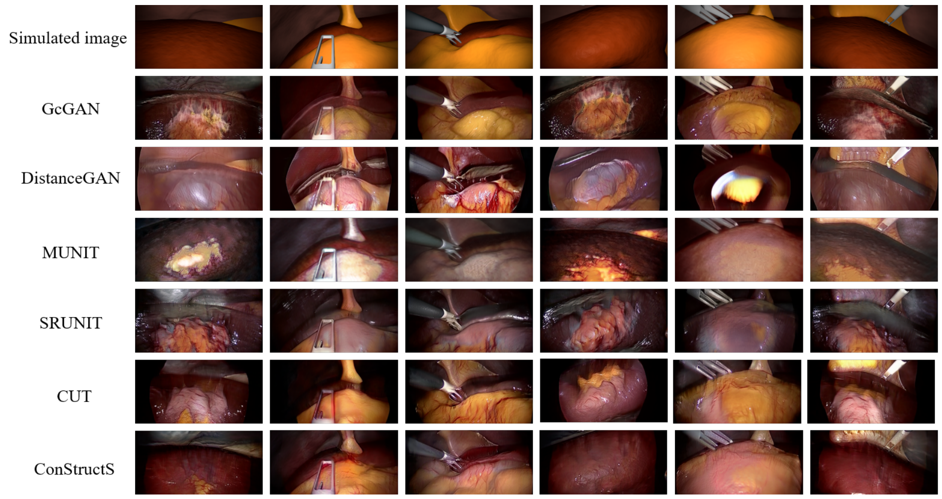

Fig. 4 Additional results from the cholecystectomy dataset.

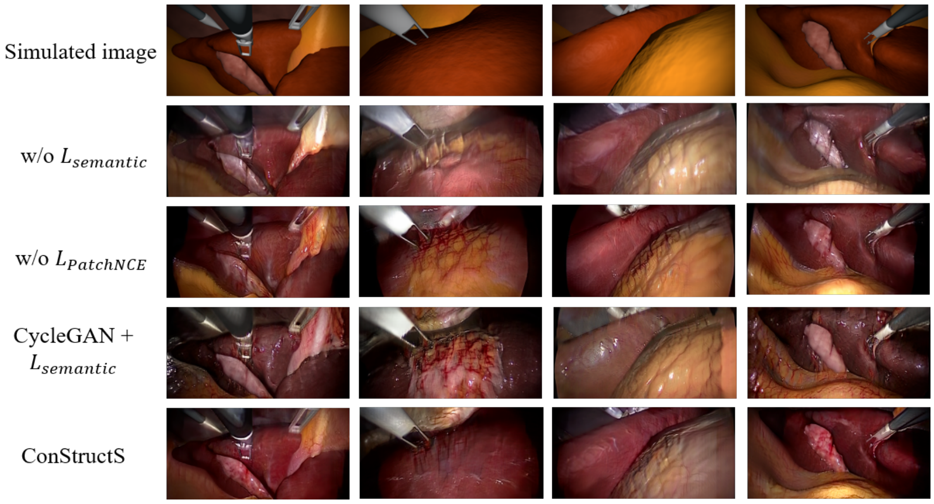

Fig. 5 Additional results from the ablation study on the cholecystectomy dataset.

and without the $\mathcal{L}_{Patch}(Y)$. The model performance deteriorates in the absence of $\mathcal{L}_{Patch}(Y)$.

5.2 Gastrectomy

The visual results depicted in Figure 6 demonstrate that ConStructS significantly mitigates semantic mismatches, particularly in regions characterized by differing specularity compared to other models. For the gastrectomy dataset, we noticed that the real images contain three extra classes compared to the synthetic domain. Mainly, there existed a class/element gauze(white) that exceeded in proportion to the other

Models	pxAcc	clsAcc	mIOU
ConStructS w/o $\mathcal{L}_{Patch}(Y)$	0.50 ± 0.06	0.41 ± 0.13	0.25 ± 0.08
ConStructS	$\mathbf{0.59 \pm 0.07}$	$\mathbf{0.44 \pm 0.12}$	$\mathbf{0.29 \pm 0.09}$

Table 2 The *consistency* evaluation results with and without the $\mathcal{L}_{Patch}(Y)$ loss.

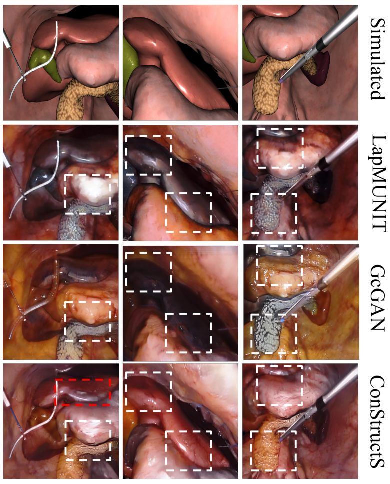

Fig. 6 Qualitative samples from the gastrectomy dataset. The white boxes highlight some regions. The red box indicates one of the failure cases of ConStructS, where a tool-like texture is mapped on the liver.

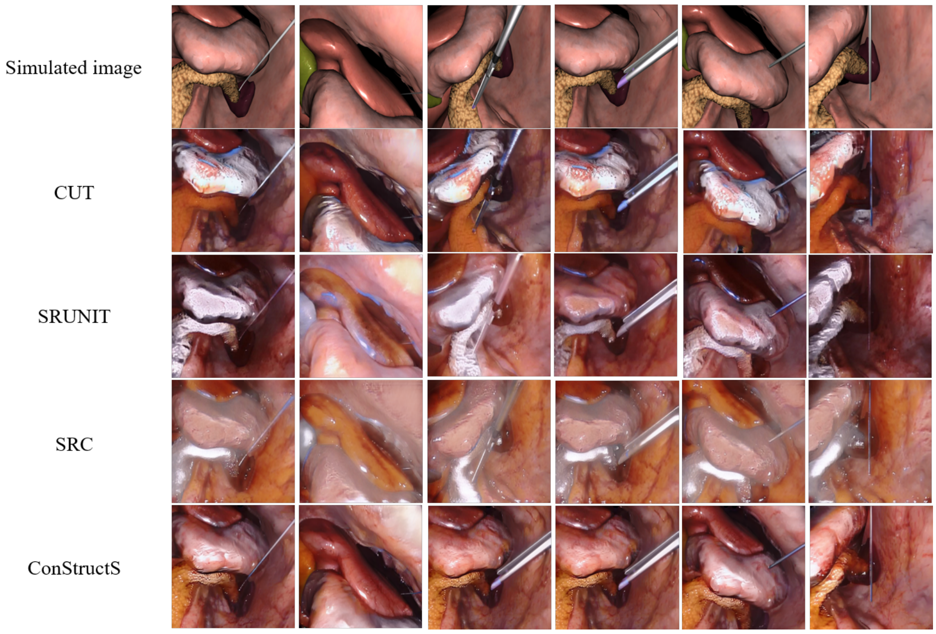

Fig. 7 Additional qualitative results from the the gastrectomy dataset.

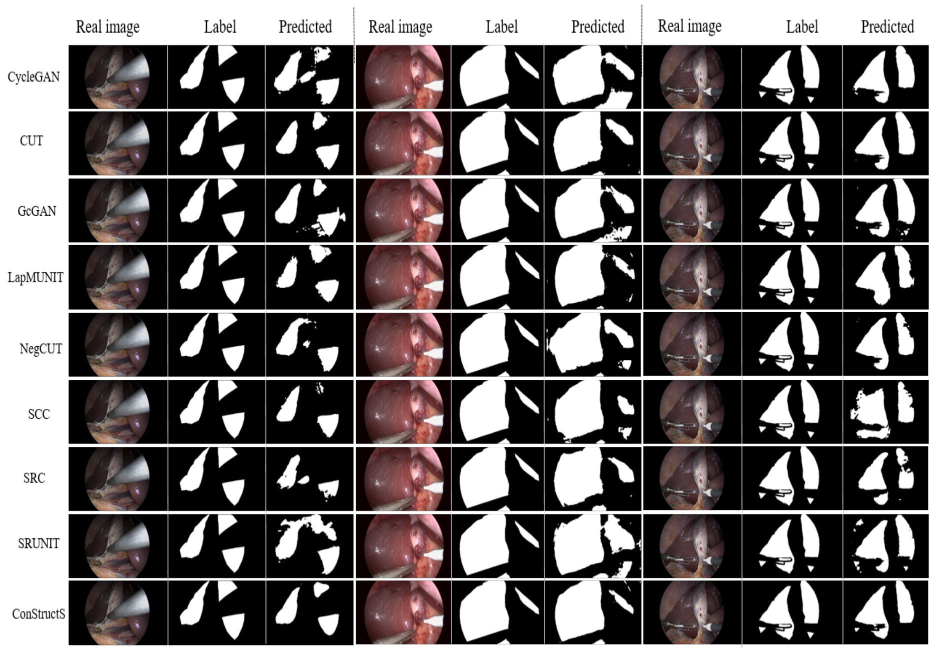

Fig. 8 The segmentation results of different models on the cholecystectomy dataset.

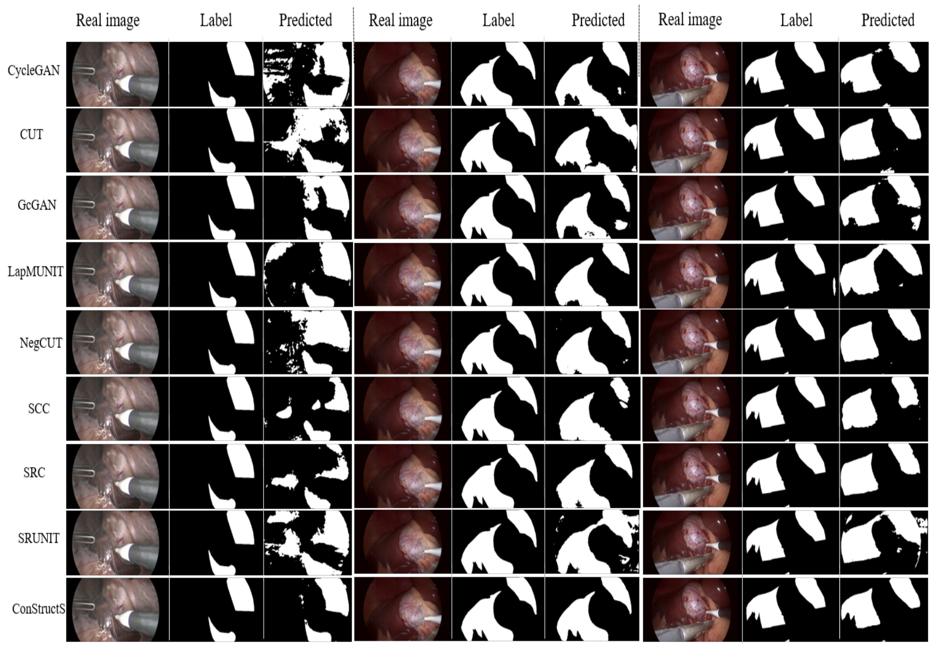

Fig. 9 Additional results from the segmentation of real images.

classes. This semantic imbalance was reflected in the translation performance, where many models mapped this texture, especially to regions with higher specularity. From the results displayed in Figure 7, we can see that ConStructS minimizes the semantic mismatch; however, few failure cases exist. The semantic imbalance leads to adverse effects on the translation quality in other models, where gauze-like texture (white) gets mapped onto the stomach.

5.3 Downstream evaluation

In Table 3, the results on *downstream* evaluation is reported. When compared to the baseline models, using only the translated images as training data from ConStructS leads to an overall improvement of 3% in dice scores. After fine-tuning this model, the data from ConStructS proves the most effective for training. Qualitative results of different models on segmentation of liver is shown in Figure 8 and Figure 9.

In Table 4 the segmentation scores of various models are shown for multi-class downstream evaluation. The translated images from the LapMUNIT [3] outperforms most the SOTA models. However, the images from the ConStructS model proves useful in improving the scores upto 6%.

5.4 Metrics

We report FID score for the different models on the cholecystectomy dataset. The FID values for both the datasets on various layers are shown in Table 5 and Table 6 respectively. The general practice is to indicate the values on the layer with 2048 features. However, here we find an effect that different layers show different values. This is one of the reasons for the adoption of different evaluation schemes in this study.

6 Contrastive learning

The useful representations in higher dimensional data can be learned by relating positive and negative pairs of data. The PatchNCE loss enforces that image should share content in patch level and not only in a global manner. For example, consider a patch corresponding to the liver on the translated image. This patch should strongly associate to the liver in the input image, more than other anatomical structures in the image. The pixel level color can also be strongly associated between the patches for better translation.

The generator of the ConStructS model consists of an encoding and a decoding part. Let G_e be the encoder of the generator. During one forward step of training, the (input) image features from various layers are readily available. This is utilized to compute the PatchNCE loss in constrast to cycle consistent methods that run the forward step through another additional generator. The translated image is passed through a small MLP network ($\mathcal{B}$) with two layers to extract the image features with the same dimension as those from the G_e. To compare these features at different spatial locations, they are computed at $\mathcal{S}$ spatial locations at $\mathcal{L}$ different layers. The query point is chosen from the translated image, while the positive point corresponds to the same location on the input image. The negative point can be chosen either from the

Training data	mean dice	
	Pre-train	Fine-tune on real
Baseline	-	0.62 ± 0.11
CycleGAN+VGG [2]	0.65 ± 0.85	0.73 ± 0.08
DRIT++ [8]	0.38 ± 0.06	0.59 ± 0.10
UGAT-IT [9]	0.36 ± 0.07	0.57 ± 0.09
DistGAN [7]	0.36 ± 0.01	0.58 ± 0.06
NEGCUT [10]	0.65 ± 0.10	0.72 ± 0.09
FeSim [11]	0.19 ± 0.90	0.27 ± 0.10
LeSim [11]	0.45 ± 0.17	0.72 ± 0.10
CycleGAN+SCC [12]	0.56 ± 0.20	0.73 ± 0.10
CUT+SCC [12]	0.38 ± 0.15	0.63 ± 0.08
CUT [1]	0.60 ± 0.12	0.71 ± 0.02
ConStructS	0.65 ± 0.21	0.84 ± 0.05

Table 3 The quantitative results for downstream eval. The mean dice scores are reported.

Models	pxAcc	clsAcc	mIOU
Baseline	0.82	0.75	0.74
CycleGAN [2]	0.84	0.76	0.76
GcGAN [8]	0.83	0.74	0.74
LapMUNIT [9]	0.85	0.76	0.74
CUT [7]	0.83	0.75	0.75
SRUNIT [10]	0.81	0.73	0.75
ConStructS	0.88	0.76	0.78

Table 4 The multi-class segmentation scores for the *downstream* evaluation.

Method	L64	L192	L768	L2048
CycleGAN [2]	2.21	8.17	0.78	136.82
GcGAN [8]	2.04	9.23	0.62	127.06
LapMUNIT [9]	0.73	3.92	0.82	167.48
CUT [7]	1.88	9.05	0.65	141.31
CUT+SCC [12]	2.89	10.02	0.68	126.25
SRUNIT [10]	3.41	10.36	0.79	138.87
SRC [11]	2.02	7.79	0.71	134.82
ConStructS	1.29	6.70	0.69	121.03

Table 5 The FID scores for the translation models on cholecystectomy dataset.

Method	L64	L192	L768	L2048
CycleGAN [2]	3.10	12.76	0.82	200.64
GcGAN [8]	1.42	5.86	0.84	193.52
LapMUNIT [9]	1.26	5.55	0.82	183.27
CUT [7]	1.75	7.40	0.89	192.11
SRUNIT [10]	3.22	11.63	0.75	207.77
SRC [11]	6.20	24.29	0.71	196.91
ConStructS	2.08	9.95	0.77	171.60

Table 6 The FID scores for the translation models on gastrectomy dataset.

same input image or from a different batch of input images. In this work, we chose the negative points from the same input image.

References

[1] Park, T., Efros, A.A., Zhang, R., Zhu, J.-Y.: Contrastive learning for unpaired image-to-image translation. In: Computer Vision–ECCV 2020: 16th European Conference, Glasgow, UK, August 23–28, 2020, Proceedings, Part IX 16, pp. 319–345 (2020). Springer

[2] Zhu, J.-Y., Park, T., Isola, P., Efros, A.A.: Unpaired image-to-image translation using cycle-consistent adversarial networks. In: Proceedings of the IEEE International Conference on Computer Vision, pp. 2223–2232 (2017)

[3] Pfeiffer, M., Funke, I., Robu, M.R., Bodenstedt, S., Strenger, L., Engelhardt, S., Roß, T., Clarkson, M.J., Gurusamy, K., Davidson, B.R., *et al.*: Generating large labeled data sets for laparoscopic image processing tasks using unpaired image-to-image translation. In: Medical Image Computing and Computer Assisted Intervention–MICCAI 2019: 22nd International Conference, Shenzhen, China, October 13–17, 2019, Proceedings, Part V 22, pp. 119–127 (2019). Springer

[4] Miyato, T., Kataoka, T., Koyama, M., Yoshida, Y.: Spectral normalization for generative adversarial networks. arXiv preprint arXiv:1802.05957 (2018)

[5] Kingma, D.P., Ba, J.: Adam: A method for stochastic optimization. arXiv preprint arXiv:1412.6980 (2014)

[6] Fu, H., Gong, M., Wang, C., Batmanghelich, K., Zhang, K., Tao, D.: Geometry-consistent generative adversarial networks for one-sided unsupervised domain mapping. In: Proceedings of the IEEE/CVF Conference on Computer Vision and Pattern Recognition, pp. 2427–2436 (2019)

[7] Tran, N.-T., Bui, T.-A., Cheung, N.-M.: Dist-gan: An improved gan using distance constraints. In: Proceedings of the European Conference on Computer Vision (ECCV), pp. 370–385 (2018)

[8] Lee, H.-Y., Tseng, H.-Y., Huang, J.-B., Singh, M., Yang, M.-H.: Diverse image-to-image translation via disentangled representations. In: Proceedings of the European Conference on Computer Vision (ECCV), pp. 35–51 (2018)

[9] Kim, J., Kim, M., Kang, H., Lee, K.: U-gat-it: Unsupervised generative attentional networks with adaptive layer-instance normalization for image-to-image translation. arXiv preprint arXiv:1907.10830 (2019)

[10] Wang, W., Zhou, W., Bao, J., Chen, D., Li, H.: Instance-wise hard negative example generation for contrastive learning in unpaired image-to-image translation. In: Proceedings of the IEEE/CVF International Conference on Computer Vision, pp. 14020–14029 (2021)

[11] Zheng, C., Cham, T.-J., Cai, J.: The spatially-correlative loss for various image

translation tasks. In: Proceedings of the IEEE/CVF Conference on Computer Vision and Pattern Recognition, pp. 16407–16417 (2021)

[12] Guo, J., Li, J., Fu, H., Gong, M., Zhang, K., Tao, D.: Alleviating semantics distortion in unsupervised low-level image-to-image translation via structure consistency constraint. In: Proceedings of the IEEE/CVF Conference on Computer Vision and Pattern Recognition, pp. 18249–18259 (2022)

[13] Jung, C., Kwon, G., Ye, J.C.: Exploring patch-wise semantic relation for contrastive learning in image-to-image translation tasks. In: Proceedings of the IEEE/CVF Conference on Computer Vision and Pattern Recognition, pp. 18260–18269 (2022)

[14] Jia, Z., Yuan, B., Wang, K., Wu, H., Clifford, D., Yuan, Z., Su, H.: Semantically robust unpaired image translation for data with unmatched semantics statistics. In: Proceedings of the IEEE/CVF International Conference on Computer Vision, pp. 14273–14283 (2021)

[15] Johnson, J., Alahi, A., Fei-Fei, L.: Perceptual losses for real-time style transfer and super-resolution. In: Computer Vision–ECCV 2016: 14th European Conference, Amsterdam, The Netherlands, October 11-14, 2016, Proceedings, Part II 14, pp. 694–711 (2016). Springer

[16] Hong, W.-Y., Kao, C.-L., Kuo, Y.-H., Wang, J.-R., Chang, W.-L., Shih, C.-S.: Cholecseg8k: a semantic segmentation dataset for laparoscopic cholecystectomy based on cholec80. arXiv preprint arXiv:2012.12453 (2020)

[17] Chen, L.-C., Zhu, Y., Papandreou, G., Schroff, F., Adam, H.: Encoder-decoder with atrous separable convolution for semantic image segmentation. In: Proceedings of the European Conference on Computer Vision (ECCV), pp. 801–818 (2018)

[18] Yoon, J., Hong, S., Hong, S., Lee, J., Shin, S., Park, B., Sung, N., Yu, H., Kim, S., Park, S., *et al.*: Surgical scene segmentation using semantic image synthesis with a virtual surgery environment. In: Medical Image Computing and Computer Assisted Intervention–MICCAI 2022: 25th International Conference, Singapore, September 18–22, 2022, Proceedings, Part VII, pp. 551–561 (2022). Springer

[19] Wada, K.: Image Polygonal annotation with Python. https://github.com/labelmeai/labelme

[20] Smith, L.N., Topin, N.: Super-convergence: Very fast training of neural networks using large learning rates. In: Artificial Intelligence and Machine Learning for Multi-domain Operations Applications, vol. 11006, pp. 369–386 (2019). SPIE
